# ^18^F-Fluorodeoxyglucose Uptake in *PDGFRA*-Mutant Gastrointestinal Stromal Tumors

**DOI:** 10.1001/jamanetworkopen.2024.56058

**Published:** 2025-01-24

**Authors:** Maria Concetta Nigro, Andrea Marchetti, Elena Rosa Fumagalli, Ida De Luca, Alexia Francesca Bertuzzi, Maria Susanna Grimaudo, Giovanni Grignani, Lorenzo D’Ambrosio, Alessandra Merlini, Giuseppe Badalamenti, Lorena Incorvaia, Alessandra Dimino, Silvia Gasperoni, Bruno Vincenzi, Stefano Fanti, Alessandro Di Federico, Davide Campana, Maria Abbondanza Pantaleo, Margherita Nannini

**Affiliations:** 1 Department of Medical and Surgical Sciences, University of Bologna, Bologna, Italy; 2 Medical Oncology Unit 2, Fondazione Istituto di Ricovero e Cura a Carattere Scientifico (IRCCS), Istituto Nazionale dei Tumori, Milano, Italy; 3 Department of Medical Oncology, Humanitas Cancer Center, IRCCS Humanitas Research Hospital, Rozzano, Milano, Italy; 4 Department of Medical Oncology, Azienda Ospedaliera Universitaria Città della Salute e della Scienza, Torino, Italy; 5 Candiolo Cancer Institute, Fondazione del Piemonte–IRCCS, Candiolo (Turin), Turin, Italy; 6 Department of Medical Oncology, University of Turin, Turin, Italy; 7 Azienda Ospedaliera Universitaria San Luigi Gonzaga University Hospital, Orbassano, Italy; 8 Department of Surgical, Oncological and Oral Sciences, Section of Medical Oncology, University of Palermo, Palermo, Italy; 9 Department of Oncology, Clinical Oncology Unit, University Hospital Careggi, Firenze, Italy; 10 Department of Medical Oncology, Campus Biomedico University of Rome, Rome, Italy; 11 Nuclear Medicine, IRCCS, Azienda Ospedaliero Universitaria Di Bologna, Bologna, Italy; 12 Department of Medical Oncology, IRCCS Azienda Ospedaliero Universitaria di Bologna, Bologna, Italy

## Abstract

**Importance:**

The D842V platelet-derived growth factor receptor α (*PDGFRA*) mutation identifies a molecular subgroup of gastrointestinal stromal tumors (GISTs), primarily resistant to standard tyrosine kinase inhibitors and with an overall more indolent behavior. Although functional imaging with ^18^F-fluorodeoxyglucose–labeled positron emission tomography ([^18^F]FDG-PET) plays a proven role in GISTs, especially in early assessment of tumor response, less is known about [^18^F]FDG uptake according to the GIST molecular subtypes.

**Objective:**

To evaluate the degree of [^18^F]FDG uptake in *PDGFRA*-mutant GISTs and better define the role of functional imaging in this rare and peculiar subset of GISTs.

**Design, Setting, and Participants:**

This multi-institutional retrospective cohort study involving 7 GIST reference centers in Italy included patients with *PDGFRA*-mutant GIST who underwent [^18^F]FDG-PET from January 1, 2000, to December 31, 2023. Data on the maximum standardized uptake value (SUVmax) of primary tumor or metastatic disease were collected.

**Exposure:**

*PDGFRA*-mutant GIST and [^18^F]FDG-PET.

**Main Outcome and Measure:**

The primary outcome was the degree of [^18^F]FDG uptake of *PDGFRA*-mutant GISTs, with a focus on the D842V-mutant subgroup. Secondary objectives were to assess the association between the degree of [^18^F]FDG uptake and main clinicopathologic features.

**Results:**

A total of 71 patients with *PDGFRA*-mutant GISTs were included in the analysis: 37 (52.1%) in the D842V subgroup (group A) and 34 (47.9%) in the non-D842V subgroup (group B). Additionally, 70 patients with *KIT* exon 11–mutant GIST served as a control group (group C). For all 141 participants, the median age at diagnosis was 59 (range, 26-89) years, and 81 patients (57.4%) were male. Overall, the median SUVmax was 4.4 (IQR, 0-10.1), while the median SUVmax for group A was 0 (IQR, 0-3.2); for group B, 3.6 (IQR, 0-5.1); and for group C, 10.1 (IQR, 5.1-13.9). The median SUVmax of *PDGFRA*-mutant GISTs was significantly lower than the median value of *KIT* exon 11–mutant GISTs (0 [IQR, 0-4.3] vs 10.1 [IQR, 5.1-14.0]; *P* < .001). Median [^18^F]FDG uptake was significantly lower in the D842V subgroup compared with the non-D842V subgroup (0 [IQR, 0-3.2] vs 3.6 [IQR, 0-5.1]; *P* = .02). Moreover, the triad of gastric primary tumor, tumor size greater than 10 cm, and SUVmax of 5.75 or less was associated with identification of *PDGFRA*-mutant GISTs.

**Conclusions and Relevance:**

In this cohort study of patients with *PDGFRA*-mutant GISTs, the D842V-mutant GISTs were associated with an overall lower [^18^F]FDG uptake compared with other GIST subgroups. Therefore, the role of functional imaging with [^18^F]FDG-PET in this subset of GISTs may be limited and should be further explored for its potential prognostic and predictive value.

## Key Points

**Question:**

What is the clinical role of positron emission tomography (PET) with ^18^F-fluorodeoxyglucose ([^18^F]FDG) in platelet-derived growth factor receptor α (*PDGFRA*)–mutant gastrointestinal stromal tumor (GIST), especially in the D842V-mutant subgroup?

**Findings:**

In this cohort study, 141 patients with GIST (71 with *PDGFRA*-mutant GIST and 70 with *KIT* exon 11–mutant GIST) who underwent [^18^F]FDG-PET were included. The median maximum standardized uptake of *PDGFRA*-mutant GISTs was significantly lower than in exon 11 *KIT*–mutant GISTs, and the median [^18^F]FDG uptake of D842V-mutant GISTs was significantly lower than in non–D842V-mutant tumors.

**Meaning:**

These results suggest that the role of functional imaging with [^18^F]FDG-PET in *PDGFRA*-mutant GISTs deserves to be explored in future prospective studies to better integrate functional imaging in clinical practice and explore its potential prognostic and predictive value.

## Teaser

This cohort study patients with platelet-derived growth factor receptor α (*PDGFRA*)-mutant GIST who underwent F 18–labeled positron emission tomography ([^18^F]FDG-PET) assess the degree of [^18^F]FDG uptake of *PDGFRA*-mutant gastrointestinal stromal tumors, with a focus on the D842V-mutant subgroup.

## Introduction

Gastrointestinal stromal tumors (GISTs) represent the most common mesenchymal neoplasm involving the gastrointestinal tract, accounting for less than 1% of all the malignant neoplasms of the digestive system.^1,2^ Although most GISTs carry activating *KIT* (OMIM 164920) mutations, which are known to confer greater sensitivity to imatinib and other tyrosine kinase inhibitors (TKIs), about 5% to 10% of GISTs harbor activating mutations of platelet-derived growth factor receptor α (*PDGFRA*) (OMIM 173490), in a mutually exclusive manner.^3,4^ This rare molecular subset of GISTs presents both different clinical behavior and variable sensitivity to TKIs, according to the exon involved and the type of mutation detected.^5^

The most common *PDGFRA* mutation is the exon 18 D842V substitution that identifies a peculiar subgroup of GISTs, with well-settled clinical and pathological features and especially known for its primary resistance to all standard therapies.^6^ In detail, D842V-mutant GISTs mainly arise from the stomach, with greater dimensions over 5 cm but low mitotic index (the fraction of tumor cells in mitosis per high-power field).^7,8,9^ They often have an epithelioid morphology and low to absent immunohistochemical positivity for *KIT* (CD117).^10,11^ From a molecular standpoint, D842V-mutant GISTs exhibit a highly uniform molecular profile when compared with *KIT*-mutant GISTs. In recent years, there has been increasing evidence supporting their greater immunogenicity.^12,13,14,15,16,17^ Finally, while they are generally characterized by an indolent behavior when localized, D842V-mutant GISTs have always been considered the “black sheep” of GISTs when metastatic, given their primary resistance to imatinib and other approved drugs.^18,19,20,21,22^ At present, the advent of avapritinib, a potent selective *KIT* and *PDGFRA* inhibitor, radically changed the prognosis of advanced D842V-mutant GISTs, becoming the new standard of care for this specific molecular subset.^1,23,24^

In clinical practice, functional imaging with positron emission tomography (PET) using ^18^F-fluorodeoxyglucose ([^18^F]FDG) plays a proven role in early assessment of GIST tumor response, especially in cases where imaging assessment is more challenging, or when early prediction of response is clinically meaningful for patient management.^1,25,26,27^ However, less is known about [^18^F]FDG uptake according to the different mutational profiles. Therefore, considering D842V-mutant GIST peculiarities detailed above, we asked whether this subset of tumors might also have a different [^18^F]FDG uptake. The aim of the present study was to investigate the degree of [^18^F]FDG uptake of *PDGFRA*-mutant GISTs, focusing on D842V mutations, for better defining the clinical role of functional imaging in this rare and peculiar subset of GIST.

## Methods

### Patients

Patients with localized and/or metastatic *PDGFRA*-mutant GIST from 7 GIST Italian reference centers in the European Reference Network on Rare Adult Cancers Network who underwent [^18^F]FDG-PET from January 1, 2000, to December 31, 2023, were retrospectively and consecutively included in this cohort study. Clinicopathologic, genomic, and maximum standardized uptake value (SUVmax) data were collected and deidentified by physicians from electronic medical records. All patients without *PDGFRA* mutations confirmed by validated molecular testing or without SUVmax data from the [^18^F]FDG-PET report were excluded from the analysis. In addition, patients with localized and/or metastatic *KIT* exon 11–mutant GISTs and known SUVmax data were included as a control group. Collected data included patient age at diagnosis, sex, primary tumor site and size, mitotic index, risk stratification for localized GISTs according to Miettinen and Lasota,^28^ molecular profiling, and disease and patient status at last contact or follow-up visit. We divided the whole cohort into 3 subgroups, according to the mutational profiling: group A, including patients with *PDGFRA* D842V–mutant GISTs; group B, including patients with *PDGFRA* non–D842V-mutant GISTs; and group C, including patients with *KIT* exon 11–mutant GISTs.

The study protocol was in conformity with the ethical guidelines of the 1975 Declaration of Helsinki. This study was approved by the local ethics committee of the S. Orsola-Malpighi Hospital in Bologna and by the institutional review boards of the other Italian participating centers. Whenever possible, patients’ confirmed written consent was obtained. All results reported are adherent to the Strengthening the Reporting of Observational Studies in Epidemiology (STROBE) reporting guideline.

The primary objective of this study was to investigate the degree of [^18^F]FDG uptake of *PDGFRA*-mutant GISTs, focusing on the D842V-mutant subgroup. Secondary objectives were to evaluate the association between the degree of [^18^F]FDG uptake and main clinicopathologic features, including extent of disease at diagnosis (localized vs advanced), primary tumor site and size, mitotic index, and Miettinen and Lasota risk class^28^; furthermore, we aimed to determine the accuracy of [^18^F]FDG uptake in distinguishing GISTs harboring the *PDGFRA* D842V mutation from those with *KIT* exon 11 mutations. In cases of multiple SUVmax measurements from different sites of disease in the same [^18^F]FDG-PET report, the highest value was considered for the analyses. In cases of multiple [^18^F]FDG-PET assessments per patient, the one with the highest SUVmax was considered for the analyses.

### Statistical Analyses

For statistical analyses, the SUVmax was considered as a continuous variable, while the molecular subtype was considered as a categorical variable. The Shapiro-Wilk test was used to assess the distribution of the variables analyzed, independent of each other. Comparisons of variables were computed using the Mann-Whitney test or the Kruskal-Wallis test, as appropriate. The Mann-Whitney test was used to compare SUVmax between all *PDGFRA*-mutant GISTs and *KIT*-mutant GISTs (control group). The Kruskal-Wallis test was used to compare the SUVmax among each subgroup (groups A, B, and C). Categorical variables were compared using Fisher exact test or χ^2^ test, when appropriate. The area under the receiver operating characteristic curve (AUROC) analysis was adopted to identify the optimal SUVmax cutoff to distinguish *PDGFRA*-mutant from *KIT* exon 11–mutant GISTs. To estimate the predictive value of SUVmax in identifying *PDGFRA*-mutant GISTs, a logistic regression analysis was used. Variables associated with the presence of a *PDGFRA* mutation in the univariate logistic regression analysis (*P* < .05) were included in the multivariable logistic regression analysis. Last, a clinical score was developed to discriminate *PDGFRA*-mutant from *KIT* exon 11–mutant GISTs.

For each analysis, cases with missing data for the analyzed variable were excluded. SUVmax data were available for every patient included in this study. A 2-sided *P* < .05 was considered statistically significant. Statistical analyses were performed using the SPSS software, version 19.0 (IBM Corporation).

## Results

A total of 159 patients with localized or metastatic *PDGFRA*-mutant GIST were identified. After data cleaning and revision, 88 patients (55.3%) were excluded due to lack of *PDGFRA* mutations confirmed by validated molecular testing or SUVmax data from [^18^F]FDG-PET.

Seventy-one patients with localized or metastatic *PDGFRA*-mutant GIST were included: 37 (52.1%) with D842V-mutant GISTs (group A) and 34 (47.9%) with non–D842V-mutant GISTs (group B). In addition, 70 patients with localized and/or metastatic *KIT* exon 11–mutant GISTs were included as a control group (group C), for a total of 141 patients. Main baseline clinicopathologic characteristics are summarized in the eTable in Supplement 1. Overall, the median age at diagnosis was 59 (range, 26-89) years, 81 patients (57.4%) were male and 60 (42.6%) were female, and 105 (74.5%) had localized disease at the diagnosis. For most patients, tumors originated from the stomach (103 [73.0%]), the primary tumor size was greater than 10 cm (61 of 134 with known data [45.5%]), and the mitotic index was greater than 5/50 per high-power field (51 of 98 with known data [52.0%]); most tumors were at high risk according to the Miettinen and Lasota classification^28^ (53 of 102 with known data [52.0%]).

**Figure 1.**
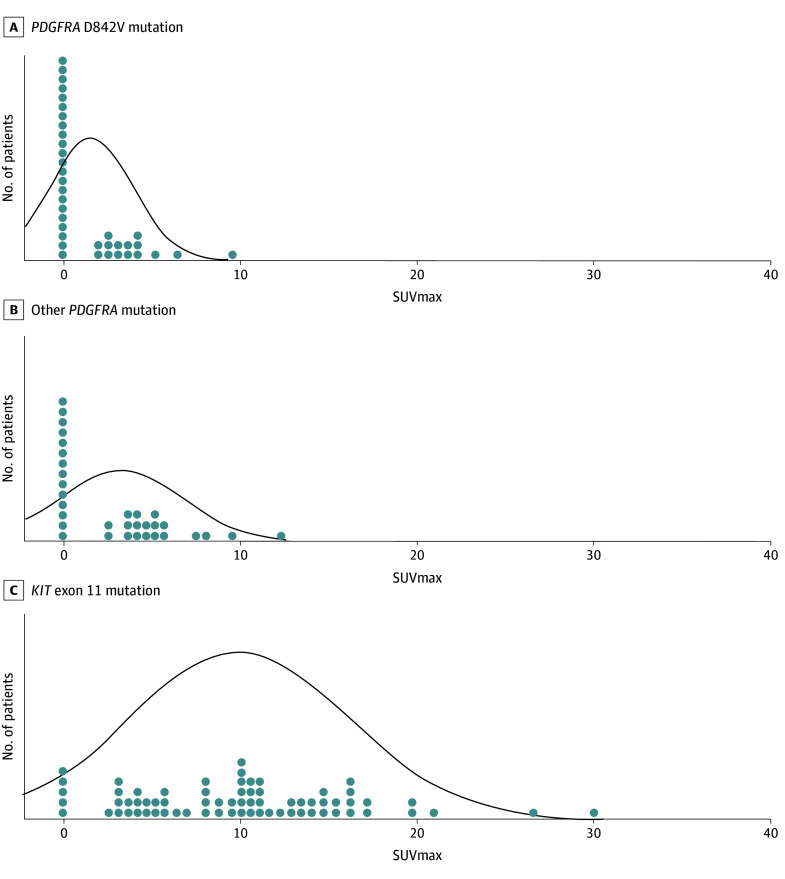
Graphic Representation of Maximum Standardized Uptake Value (SUVmax) Distribution and Median SUVmax of the 3 Molecular Subgroups of Gastrointestinal Stromal Tumors A, The median SUVmax was 0. B, The median SUVmax was 3.6. C, The median SUVmax was 10.1. The curves represent the SUVmax distribution in each molecular subgroup. *PDGFRA* indicates platelet-derived growth factor receptor α.

**Figure 2.**
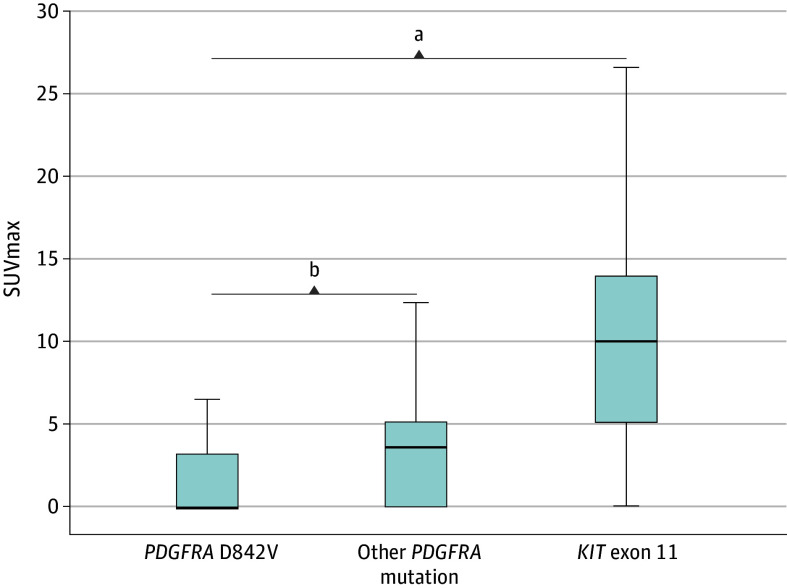
Association Between ^18^F-Fluorodeoxyglucose Uptake and Molecular Pattern of Gastrointestinal Stromal Tumors Horizontal black bars indicate the median; error bars forming the rectangle represent the interquartile range, while the upper error bar represents the maximum standardized uptake value (SUVmax) that is not an outlier and the lower error bar represents the minimum SUVmax that is not an outlier. *PDGFRA *indicates platelet-derived growth factor receptor α. ^a^The median SUVmax of *PDGFRA*-mutant GISTs was significantly lower than that of *KIT* exon 11–mutant GISTs (0 [IQR, 0-4.3] vs 10.1 [IQR, 5.1-14.0]; *P* < .001). ^b^Within *PDGFRA*-mutant gastrointestinal stromal tumors (GISTs), median SUVmax was significantly lower in D842V-mutant GISTs compared with non–D842V-mutant ones (0 [IQR, 0-3.2] vs 3.6 [IQR, 0-5.1]; *P* = .02).

SUVmax measurements were obtained from the primary tumor in 74 patients (52.5%) (20 in group A, 20 in group B, and 34 in group C) and from metastatic lesions in 67 (47.5%) (19 with D842V mutation, 13 with non-D842V mutation, and 35 with *KIT* mutation). Considering the whole population of 141 patients, the global median SUVmax was 4.4 (IQR, 0-10.1), while the median SUVmax for group A was 0 (IQR, 0-3.2), for group B was 3.6 (IQR, 0-5.1), and for group C was 10.1 (IQR, 5.1-13.9) (Figure 1). The median SUVmax of *PDGFRA*-mutant GISTs was significantly lower than that of *KIT* exon 11–mutant GISTs (0 [IQR, 0-4.3] vs 10.1 [IQR, 5.1-14.0]; *P* < .001) (Figure 2). Of note, within *PDGFRA*-mutant GISTs, median SUVmax was significantly lower in D842V-mutant GISTs compared with non–D842V-mutant ones (0 [IQR, 0-3.2] vs 3.6 [IQR, 0-5.1]; *P* = .02).

Because significant differences in [^18^F]FDG SUVmax were observed among GIST molecular subgroups, we used an AUROC analysis to identify an optimal SUVmax cutoff able to distinguish *PDGFRA-* from *KIT* exon 11–mutant GISTs. The SUVmax cutoff of 5.75 resulted in the most accurate discrimination against the 2 molecular GIST subgroups (sensitivity, 71.4%; specificity, 90.1%), with an AUROC of 0.870 (95% CI, 0.811-0.929; *P* = .001) (eFigure in Supplement 1). Next, we explored whether clinicopathologic features of GISTs were associated with the degree of [^18^F]FDG uptake, using the identified SUVmax cutoff of 5.75. Compared with GISTs with an SUVmax greater than 5.75 (n = 57), those with an SUVmax of 5.75 or less (n = 84) were more likely to be localized in the stomach (72 [85.7%] vs 31 [54.4%]; *P* < .001), to have a lower mitotic index (≤5/50 per high-power field in 37 of 60 [61.7%] vs 10 of 38 [26.3%] with data available; *P* = .001), and less likely to belong to the Miettinen and Lasota high-risk class ^28^ (25 of 66 [37.9%] vs 28 of 36 [77.8%] with data available; *P* = .002), while having no significant difference in stage of disease and no association with size of the primary tumor at diagnosis (Table 1).

**Table 1.** SUVmax Cutoff According to Clinicopathologic Features

Characteristic	Patients, No. (%)			*P* value
	SUVmax		All (N = 141)	
	≤5.75 (n = 84)	>5.75 (n = 57)		
Disease stage				
Localized	68 (81.0)	37 (64.9)	105 (74.5)	.05
Advanced or metastatic	16 (19.0)	20 (35.1)	36 (25.5)	
Primary tumor site				
Digiunum or duodenum	2 (2.4)	7 (12.3)	9 (6.4)	.001
Esophagus	1 (1.2)	0	1 (0.7)	
Ileum	7 (8.3)	15 (26.3)	22 (15.6)	
Rectum	2 (2.4)	3 (5.3)	5 (3.5)	
Stomach	72 (85.7)	31 (54.4)	103 (73.0)	
Extragastrointestinal	0	1 (1.8)	1 (0.7)	
Primary tumor size, cm^a^				
<5	14 (17.3)	10 (18.9)	24 (17.9)	.92
>10	38 (46.9)	23 (43.4)	61 (45.5)	
5-10	29 (35.8)	20 (37.7)	49 (36.6)	
Mitotic index, HPF^b^				
≤5/50	37 (61.7)	10 (26.3)	47 (48.0)	.001
>5/50	23 (38.3)	28 (73.7)	51 (52.0)	
Classification of risk^c^				
Very low	8 (12.1)	1 (2.8)	9 (8.8)	.002
Low	14 (21.2)	3 (8.3)	17 (16.7)	
Intermediate	19 (28.8)	4 (11.1)	23 (22.5)	
High	25 (37.9)	28 (77.8)	53 (52.0)	

Abbreviations: HPF, high-power field; SUVmax, maximum standardized uptake value.

^a^ Three patients were missing for SUVmax of 5.75 or greater and 4 for SUVmax greater than 5.75.

^b^ Twenty-four patients were missing for SUVmax of 5.75 or greater and 49 for SUVmax greater than 5.75.

^c^ According to risk-stratification criteria by Miettinen and Lasota.^28^ Eighteen patients were missing for SUVmax of 5.75 or greater and 21 for SUVmax greater than 5.75.

Considering the known genotype-phenotype correlation in GISTs, we then evaluated the potential predictive value of a combined functional-clinical-pathological pattern analysis in discriminating the *PDGFRA*-mutant from the *KIT* exon 11–mutant subgroup using a logistic regression analysis (Table 2). No association was found between genotype and sex (odds ratio [OR] for male sex, 0.91 [95% CI, 0.47-1.78]; *P* = .79), disease stage at diagnosis (OR for advanced stage, 0.62 [95% CI, 0.29-1.34]; *P* = .23), or mitotic index (OR, 0.50 [95% CI, 0.22-1.12]; *P* = .09). Conversely, the SUVmax cutoff of 5.75 was able to discriminate the GIST genotype both in a univariate (OR, 0.04 [95% CI, 0.02-0.11]; *P* < .001) and a multivariate model (OR, 0.03 [95% CI, 0.01-0.10]; *P* < .001), confirming an independent association between an SUVmax of 5.75 or less and the presence of a *PDGFRA* mutation. As expected, in the same multivariable model, we observed an independent association between the GIST genotype and tumor primary site (OR for ileum vs stomach, 0.11 [95% CI, <0.02 to 0.57]; *P* = .008]; OR for other site vs stomach, 0.08 [95% CI, 0.01-0.55]; *P* = .01]). The same analysis also revealed an independent association between GIST genotype and tumor primary size (OR for >10 vs <5 cm, 7.91 [95% CI, 1.90-32.84]; *P* = .004), confirming the association among the presence of a *PDGFRA* mutation, stomach as the primary site, and a greater tumor size. Next, we combined these 3 variables that resulted in an independent association with GIST genotype (SUVmax ≤5.75, gastric location, and tumor size >10 cm) in a clinical score to enhance their accuracy in discriminating *PDGFRA*-mutant from *KIT* exon 11–mutant GISTs, assigning 1 point to each of them (Table 3). Interestingly, we observed that 31 of 33 patients (93.9%) with GISTs and a score of 3 of 3 belonged to the *PDGFRA-*mutant group. On the contrary, all the 19 patients with GISTs with a score of 0 of 3 belonged to the *KIT* exon 11–mutant subgroup.

**Table 2.** Univariate and Multivariate Logistic Regression Analysis to Determine Parameters Associated With the Ability to Predict *PDGFRA* Mutations

Characteristic	Univariate		Multivariate	
	OR (95% CI)	*P* value	OR (95% CI)	*P* value
SUVmax >5.75	0.04 (0.02-0.11)	<.001	0.03 (0.01-0.10)	<.001
Sex (male)	0.91 (0.47-1.78)	.79	NA	NA
Stage of disease (advanced)	0.62 (0.29-1.34)	.23	NA	NA
Primary site of tumor				
Ileum vs stomach	0.09 (0.025-0.32)	<.001	0.11 (<0.02-0.57)	.008
Other site vs stomach	0.08 (0.02-0.37)	.001	0.08 (0.01-0.55)	.01
Tumor dimension, cm				
5-10 vs <5	1.92 (0.69-5.31)	.21	3.80 (0.91-15.75)	.06
>10 vs <5	3.30 (1.22-8.93)	.02	7.91 (1.90-32.84)	.004
Mitotic index, HPF				
≤5/50 vs >5/50	0.50 (0.22-1.12)	.09	NA	NA

Abbreviations: HPF, high-power field; NA, not applicable; OR, odds ratio; *PDGFRA*, platelet-derived growth factor receptor α; SUVmax, maximum standardized uptake value.

**Table 3.** Combined Functional-Clinical-Pathological Score in Identifying the *PDGFRA*-Mutant Gastrointestinal Stromal Tumors

Score^a^	Patients, No. (%)		No. of patients overall
	*PDGFRA* mutations	*KIT* mutations	
0 of 3	0	19 (100)	19
1 of 3	5 (17.2)	24 (82.8)	29
2 of 3	35 (58.3)	25 (41.7)	60
3 of 3	31 (93.9)	2 (6.1)	33

Abbreviation: *PDGFRA*, platelet-derived growth factor receptor α.

^a^ Scores include gastric localization (1), size greater than 10 cm (1), and maximum standardized uptake value of 5.75 or less (1).

## Discussion

As hypothesized, this cohort study found that *PDGFRA*-mutant GISTs had a median [^18^F]FDG uptake significantly lower than the median value observed in *KIT* exon 11–mutant GISTs. Notably, we also found that within the whole *PDGFRA*-mutant subgroup, *PDGFRA* D842V–mutant GISTs presented a median [^18^F]FDG uptake significantly lower compared with that of *PDGFRA* non–D842V mutant ones. This metabolic pattern may be associated with the peculiar clinicopathologic features of this rare molecular subtype of GIST, generally characterized by gastric localization, size greater than 5 cm, and low mitotic index. In line with these data, we also found an association between an SUVmax threshold of 5.75 and both mitotic index and nongastric localization, whereas no association with primary tumor size was found. On this basis, we then evaluated the potential predictive value of a combined functional-clinical-pathological pattern analysis pointing out the triad of gastric localization, primary tumor size greater than 10 cm, and SUVmax of 5.75 or less that presented an accuracy of 93.9% in identifying *PDGFRA*-mutant cases in our study population.

As is well known, functional imaging with [^18^F]FDG-PET is a valuable tool for early assessment of GIST tumor response. This is particularly relevant when there is uncertainty regarding response, or when early prediction of tumor response guides the clinical decision-making.^1,25,26,27^ This approach has been widely applied to almost all oncogene-addicted solid tumors treated with TKIs, making GISTs a paradigm also for radiological and/or metabolic response assessment in the era of precision oncology.^29,30^ Indeed, functional imaging may help in identifying those patients who are likely to benefit from a molecular targeted therapy. Conversely, it can also help in identifying those patients who may have tumors primarily resistant to treatment, although the prognostic value of the degree of metabolic response is still debated.^27,31^ Moreover, positive correlations between [^18^F]FDG uptake and several clinicopathologic features, such as tumor size, mitotic count, and risk stratification, have been found in some case series,^32,33,34^ suggesting that functional imaging may be also useful for preoperative assessment of GIST malignant potential. Consistently with these observations, high expression levels of glucose transporter 1 (*GLUT1*), hexokinase 1 (*HK1*), and lactate dehydrogenase A (*LDHA*) in high-risk GISTs have been reported, likely explaining their glucose-prone metabolism.^32^ Nevertheless, to our knowledge, the association between [^18^F]FDG uptake and GIST molecular profiling has not been thoroughly investigated.

GISTs are widely recognized as a heterogenous set of different entities, according to their molecular profile, which affects clinical presentation, aggressiveness, prognosis, and sensitivity to standard treatments. In this scenario, D842V *PDGFRA*-mutant GISTs represent an intriguing molecular subset of GISTs, with distinctive clinicopathologic and biological features that might affect their glucose metabolism.

To our knowledge, this is the first and largest study on functional imaging specifically focused on *PDGFRA*-mutant GISTs. The observation of lower [^18^F]FDG uptake in the *PDGFRA* D842V-mutant GISTs subgroup compared to with other GIST subgroups is particularly relevant for treatment response evaluation, given the recent advent of avapritinib as a new standard of care for this subset of GISTs, and might allow reduction of costs and radiation exposure. Moreover, the proposed triad of gastric localization, primary tumor size greater than 10 cm, and low SUVmax may have a clinical utility in identifying *PDGFRA*-mutant GISTs, for better tailoring both molecular assessment and clinical decision-making. However, it is important to underline that this triad should not replace molecular analysis, which remains essential unless there is a lack of adequate specimen sample and/or a new histological sampling is challenging or potentially harmful for the patient.

### Limitations

This cohort study has limitations, mainly related to its retrospective nature and to the rarity of the GIST molecular subtype that has been considered. Indeed, to collect the greatest number of patients who met the inclusion criteria, the recruitment period was around 20 years. As a consequence, the SUVmax data are not fully comparable. Besides, the multicentric design of the study has surely contributed to this nonuniformity in methods adopted. However, the relevance of this cohort study is that, despite the heterogeneity of the study population, all findings are overall consistent.

## Conclusions

In this cohort study of *PDGFRA*-mutant GISTs, the *PDGFR* D842V-mutant GISTs subgroup presented an overall lower [^18^F]FDG uptake compared to with other GIST subgroups. Therefore, the clinical role of functional imaging with [^18^F]FDG-PET in *PDGFRA* D842V–mutant GISTs could be limited by their overall low [^18^ F]FDG-uptake. Despite some limitations related to the present study’s retrospective nature, the evaluation of [^18^F]FDG uptake within each molecular GIST subgroup deserves to be explored in future prospective studies to better integrate functional imaging in clinical practice and explore its potential prognostic and predictive value.

## Notes

Supplement 1. **eTable.** Patients’ Clinical and Pathological Features **eFigure**. ROC Curve Used to Define the Most Reliable SUVmax Cutoff in Terms of Sensitivity and Specificity

Supplement 2. **Nonauthor Collaborators.** Nonauthor Members of Tumori Rari Bologna

Supplement 3.
Data Sharing Statement
