# Supplementary material for: 18F-Fluorodeoxyglucose Uptake in PDGFRA-Mutant Gastrointestinal Stromal Tumors
Source: JAMA Netw Open. 2025 Jan 24;8(1):e2456058. doi: 10.1001/jamanetworkopen.2024.56058 (PMC11762236; doi:10.1001/jamanetworkopen.2024.56058)
Supplement: Supplement 1. — eTable. Patients’ Clinical and Pathological Features eFigure. ROC Curve Used to Define the Most Reliable SUVmax Cutoff in Terms of Sensitivity and Specificity [file jamanetwopen-e2456058-s001.pdf]

Supplementary Online Content

Nigro MC, Marchetti A, Fumagalli ER, et al; Tumori Rari Bologna. ^{18}F-fluorodeoxyglucose uptake in *PDGFRA*-mutant gastrointestinal stromal tumors. *JAMA Netw Open*. 2025;8(1):e2456058. doi:10.1001/jamanetworkopen.2024.56058

eTable. Patients' Clinical and Pathological Features

eFigure. ROC Curve Used to Define the Most Reliable SUVmax Cutoff in Terms of Sensitivity and Specificity

This supplementary material has been provided by the authors to give readers additional information about their work.

© 2025 Nigro MC et al. *JAMA Network Open*.

eTable. Patients' Clinical and Pathological Features

Characteristic	Total<br>141	*PDGFRα* D842V (Group A)<br>37	*PDGFRα* non D842V (Group B)<br>34	*KIT*- Ex 11 (Group C)<br>70	P
	**Pt, N (%)**	**Pt, N (%)**	**Pt, N (%)**	**Pt, N (%)**	
**Gender**					
Male	81 (57.4)	26 (70.3)	14 (41.2)	41 (58.6)	P=0.04
Female	60 (42.6)	11 (29.7)	20 (58.2)	29 (41,4)	
**Disease stage at diagnosis**					
Localized	105 (74.5)	29 (78.4)	27 (79.4)	49 (70.0)	nss
Advanced	36 (25.5)	8 (21.6)	7 (20.6)	21 (30.0)	
**Primary tumor site**					
Stomach	103 (73.0)	34 (91.9)	32 (94.4)	37 (52.8)	
Ileum	22 (15.6)	1 (2.7)	2 (5.6)	19 (27.2)	
Rectum	5 (3.5)	1 (2.7)	0	4 (5.8)	P <0.001
Oesophagus	1 (0.7)	1 (2.7)	0	0	
Duodenum	9 (6.5)	0	0	9 (12.8)	
Extra-GI	1 (0.7)	0	0	1 (1.4)	
**Primary tumor size, cm**					
<5	24 (17.9)	4 (10.8)	4 (12.1)	16 (25.0)	
5-10	49 (36.6)	13 (35.1)	11 (33.4)	25 (39.1)	nss
>10	61 (45.5)	20 (54.1)	18 (54.5)	23 (35.9)	
Unknown	7	-	1	6	
**Mitotic index, HPF**					
≤5/50	47 (48.0)	15 (50.0)	16 (61.5)	16 (38.1)	
>5/50	51 (52.0)	15 (50.0)	10 (38.5)	26 (61.9)	P=0.04
Unknown	43	7	8	28	
**Class of risk**[a]					
Very low	9 (8.8)	3 (9.3)	1 (3.6)	5 (11.9)	
Low	17 (16.7)	6 (18.8)	7 (25.0)	4 (9.5)	
Intermediate	23 (22.5)	9 (28.1)	11(39.3)	3 (7.2)	P<0.001
High	53 (52)	14 (43.8)	9 (32.1)	30 (71.4)	
Unknown	39	5	6	28	

[a]*According to Miettinen's criteria for risk stratification (2006) [28]*

Ex = exon; HPF = high-power field; N = number; nss = not statistically significant; *PDGFRa* = platelet-derived growth factor receptor-ɑ; pt =patient.

© 2025 Nigro MC et al. *JAMA Network Open*.

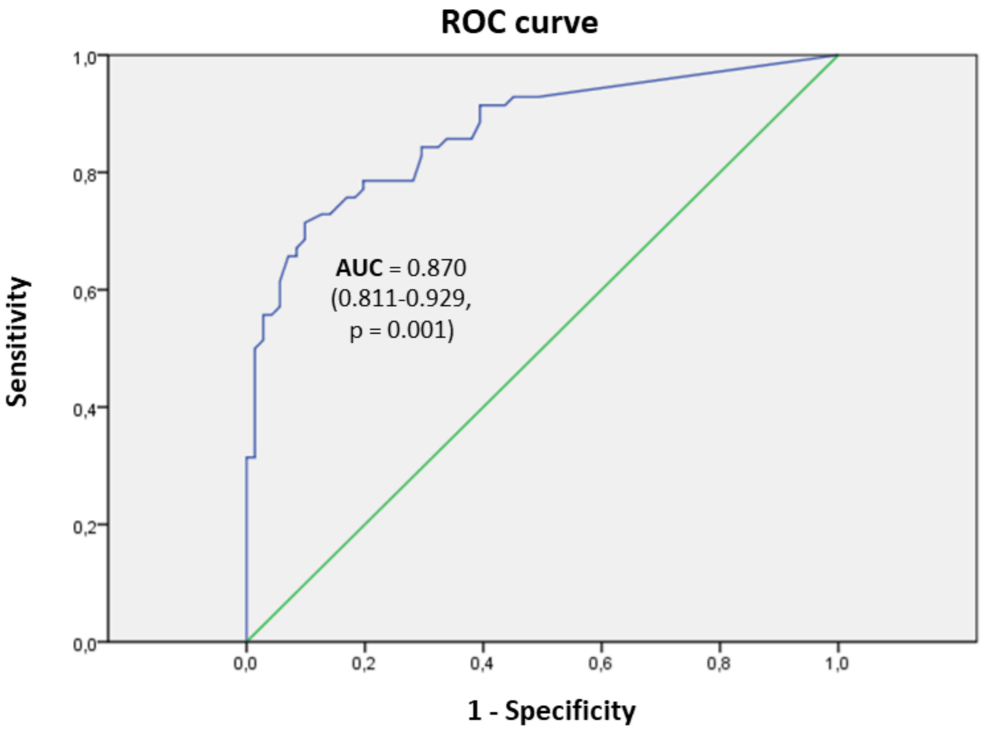

eFigure. ROC Curve Used to Define the Most Reliable SUVmax Cutoff in Terms of Sensitivity and Specificity

© 2025 Nigro MC et al. *JAMA Network Open*.
