# Supplementary material for: 18F-Fluorodeoxyglucose Uptake in PDGFRA-Mutant Gastrointestinal Stromal Tumors
Source: JAMA Netw Open. 2025 Jan 24;8(1):e2456058. doi: 10.1001/jamanetworkopen.2024.56058 (PMC11762236; doi:10.1001/jamanetworkopen.2024.56058)
Supplement: Supplement 2. — Nonauthor Collaborators. Nonauthor Members of Tumori Rari Bologna [file jamanetwopen-e2456058-s002.pdf]

Supplemental Online Content: Nonauthor Collaborators
*First name, last name, and suffix (if applicable) are required and will appear in PubMed.

*Group Name(s): Tumori Rari Bologna							
***First Name and Middle Initial(s)**	***Last Name**	***Suffix (eg, Jr, III)**	Academic Degrees	Institution	Location (city, state/province, country)	Role or Contribution, eg, chair, principal investigator	Group (if more than 1 Group listed in the byline) and/or Subgroup (eg, Steering Committee)
Annalisa	Astolfi		PhD	University of Bologna	Bologna, Italy	Sub-investigator (molecular biology)	Tumori Rari Bologna
Alice	Costa		Dr.	IRCCS Azienda Ospedaliero-Universitaria di Bologna	Bologna, Italy	Sub-investigator (molecular biology)	Tumori Rari Bologna
Manuela	Ianni		PhD	IRCCS Azienda Ospedaliero-Universitaria di Bologna	Bologna, Italy	Study coordinator	Tumori Rari Bologna
Massimo	Del Gaudio		MD	IRCCS Azienda Ospedaliero-Universitaria di Bologna	Bologna, Italy	Sub-investigator (surgery)	Tumori Rari Bologna
Dario	de Biase		Prof.	University of Bologna	Bologna, Italy	Sub-investigator (molecular biology)	Tumori Rari Bologna
Maria Giulia	Pirini		MD	IRCCS Azienda Ospedaliero-Universitaria di Bologna	Bologna, Italy	Sub-investigator (pathology)	Tumori Rari Bologna
