# Supplementary material for: 18F-Fluorodeoxyglucose Uptake in PDGFRA-Mutant Gastrointestinal Stromal Tumors
Source: JAMA Netw Open. 2025 Jan 24;8(1):e2456058. doi: 10.1001/jamanetworkopen.2024.56058 (PMC11762236; doi:10.1001/jamanetworkopen.2024.56058)
Supplement: Supplement 3. — Data Sharing Statement [file jamanetwopen-e2456058-s003.pdf]

Data Sharing Statement

Nigro. ^{18}F-Fluorodeoxyglucose Uptake in PDGFRA-Mutant Gastrointestinal Stromal Tumors. *JAMA Netw Open*. Published January 24, 2025. doi:10.1001/jamanetworkopen.2024.56058

Data
Data available: Yes
Data types: Deidentified participant data
How to access data: https://commons.datacite.org/doi.org/10.5281/zenodo.12686794
When available: beginning date: 07-08-2024

Supporting Documents
Document types: None

Additional Information
Who can access the data: Anyone requesting the data
Types of analyses: Dataset
Mechanisms of data availability: With investigator support
